# Climatic irregular staircases: generalized acceleration of global warming

**DOI:** 10.1038/srep19881

**Published:** 2016-01-27

**Authors:** Bernard De Saedeleer

**Affiliations:** 1 Belgian National Fund of Scientific Research (F.R.S.-FNRS), Belgium; 2 Earth and Life Institute, Georges Lemaître Centre for Earth and Climate Research, Université catholique de Louvain, Louvain-la-Neuve, Belgium

## Abstract

Global warming rates mentioned in the literature are often restricted to a couple of arbitrary periods of time, or of isolated values of the starting year, lacking a global view. In this study, we perform on the contrary an exhaustive parametric analysis of the NASA GISS LOTI data, and also of the HadCRUT4 data. The starting year systematically varies between 1880 and 2002, and the averaging period from 5 to 30 yr — not only decades; the ending year also varies . In this way, we uncover a whole unexplored space of values for the global warming rate, and access the full picture. Additionally, stairstep averaging and linear least squares fitting to determine climatic trends have been sofar exclusive. We propose here an original hybrid method which combines both approaches in order to derive a new type of climatic trend. We find that there is an overall acceleration of the global warming whatever the value of the averaging period, and that 99.9% of the 3029 Earth’s climatic irregular staircases are rising. Graphical evidence is also given that choosing an El Niño year as starting year gives lower global warming rates — except if there is a volcanic cooling in parallel. Our rates agree and generalize several results mentioned in the literature.

Many different values are mentioned in the scientific literature for estimating the rate of change of Earth’s global surface temperature, based on observations. First of all, there is widespread evidence that global temperature is overall rising for the last century, so that the global warming rate (GWR) is mainly positive. Additionally, there are many indicators which show that climate change is even accelerating^1^,^2^. But when examining the three gaps between the last four decades on temperature graphs like [3, Fig. SPM 1a), p. 6], the GWR seems rather to be constant (+0.17 °C/dec). In parallel, there is since 1998 an ongoing so-called ‘warming hiatus’^4^ (near-to-null GWR) for which scientific explanations have began to be provided. This slowdown in surface temperature rise^5^,^6^,^7^ is an artifact due to the fact that the ‘missing heat’ in the atmosphere^8^,^9^ is being trapped in the ocean instead^10^,^11^. The objective deductive statement is that the GWR is intrinsically highly variable.

This diversity of GWR is due to two intrinsic factors: on the one hand to the climate’s physics itself (the natural internal variability of climate [3, Box 2.5, Table 1, p. 233–235] and the anthropogenic GHG emissions forcing, mainly carbon dioxide^12^) — a reality on which we have a limited control, and on the other hand to the method used to derive these rates, on which we have a full direct control.

In order to extract the long-term climatic trend from the annual mean observations, two approaches may be followed. The first approach consists in modeling each fast natural component (with period typically lesser than 5 yr) of climate variability like in [Ref. 13], and then to subtract all of them from the full temperature signal. What is thus remaining is the long-term trend, but still sullied by unmodeled weather fluctuations anyway. The second approach is to perform some averaging process of the temperature signal over several years. In this article, we use the stairstep averaging approach because it has the advantage of very efficiently removing all higher frequency climatic noise at once, which allows to access directly the long-term trend in the climatic signal. Decadal averages are usually considered, but what happens if we change the averaging period?

Furthermore, the starting years chosen to perform the analyses are usually fixed to a few round values aligned with decades (e.g. 1880, 1901, 1901, 1951 and 1979 respectively for the five periods of analysis considered in [3, Table 2.7, p. 193]). But what happen if we allow also the starting year to vary continuously on a wider range? By performing a systematic exploration of that two-parameters space, this study provides a unifying view of all possible values for the GWR. The effect of a varying ending year is also considered.

In parallel, linear regression by way of least squares trends [3, Box 2.2, Fig. 1, p. 180] are used in the literature in order to compute climate trends. But there is no reason, in our opinion, why the stairstep averaging and the linear regression approaches should be exclusive. Hence, in this paper, we also propose an original way of combining the stairstep averages with linear regression that will be called the *hybrid method* herein. This method will allow us to derive a new type of climatic trend.

In this paper, we show by the parametric analysis performed using the proposed hybrid method that *(i)* there is an overall acceleration of the global warming *whatever the value of the averaging period*; that *(ii)* 99.9% of the 3029 Earth’s climatic irregular staircases are rising; and that *(iii)* shorter-term local minima in GWR are achieved when choosing an El Niño year as a starting year *S* — except if there is a volcanic cooling in parallel.

## Data

The global mean surface temperature (GMST) anomaly is estimated from several observational data sets (GISS LOTI, HadCRUT4^14^ or MLOST^15^ data sets) which exhibit very similar evolutions [3, Box 2.5, Table 1, p. 233–235]. Hence, in order to perform the comprehensive parametric analysis and illustrate the new hybrid concept, we use here mainly one single time series, for the sake of clarity.

As we are interested in studying the rate of surface temperature rise for the whole planet Earth at scales above the year, data has to be somehow averaged over space and time. One of the most authoritative source of data in this respect is the NASA GISS, which releases the most updated datasets (GHCN-v3 is used herein) by appropriate techniques. In the following, we will use global-mean and annual means data, which are available at http://data.giss.nasa.gov/gistemp/.

**Figure 1:**
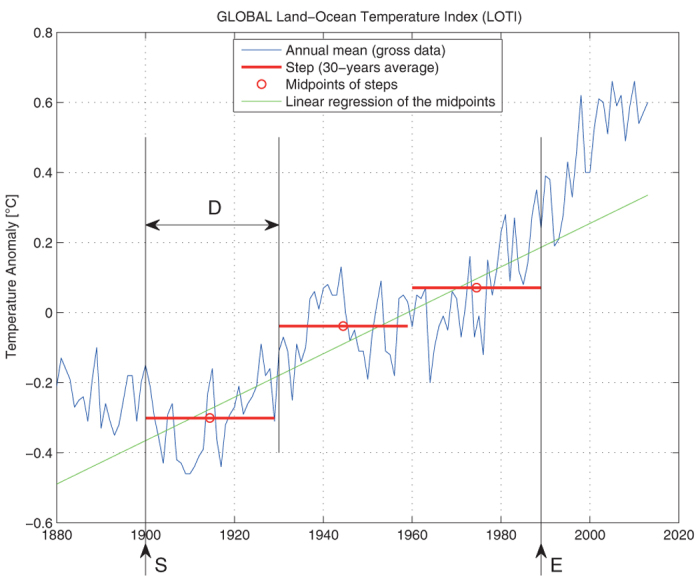
Definition of the three variables used for the parametric analysis: starting year (*S*), ending year (*E*) and duration of the step (*D*), which is the averaging period. This defines a *climatic staircase* for a particular value of the (*S*, *D*, *E*) set. The data is the NASA GISS global surface temperature LOTI index (anomalies with respect to the period 1951–1980). The proposed hybrid approach combines averages and linear regression by least squares fitting, and is illustrated here for one specific set of values: *S* = 1900, *E* = 1989 and *D* = 30 yr. The GWR is then defined as the slope of the linear regression through the midpoints of the steps.

More precisely, we use the combined land-surface air and sea-surface water temperature anomalies (land-ocean temperature index, LOTI), because it is a quantity which is more representative of the global mean trends than the land-surface air temperature (LSAT) anomalies only. This is due to the fact that the oceans do undoubtedly play a major role, regarding to their enormous heat storage capacity^16^, and to their large spatial extension (oceans that cover about two thirds of the earth’s surface). The data are temperature *anomalies*, i.e. deviations from the reference period 1951–1980, for which the best estimate for absolute global mean is 14.0 °C. This data set has already been extensively described and plotted e.g. in [17, Fig. 3] and [18, Fig. 1]. The study has also been performed on a second time series, the HadCRUT4 data set, and yields similar results as for NASA GISS (the graphs cannot be distinguished by eye).

## Method

Once the data selected, the question arises of choosing a value for the duration of the period on which the average is performed, as many different timescales are involved in the very definition of climate given in the glossary of [3, p. 1450]. Decadal averages have been successfully used in the last IPCC report [3, Fig. SPM 1a), p. 6], to analyze observed globally combined land and ocean surface temperature anomalies for the period 1850–2012. Periods of 5 yr (in order to remove ENSO and volcanoes effects) and 11 yr (in order to remove the solar cycle effect) have been used in [17, Fig. 21] to compute running averages. Periods of 15 yr have also been used in order to compute the last 15 years trend [3, SPM, p. 3]. In parallel, “the classical period for averaging these variables is 30 years, as defined by the World Meteorological Organization” [3, p. 1450]. So we ask the following question: how does the GWR value change if we allow the averaging period to vary between 5 and 30 yr? And also, what is the effect of changing the starting year of the analysis? Or the ending year?

In order to explore systematically the whole possible space within this framework, we define three ad hoc variables: the *starting year* (*S*), the *ending year* (*E*) and the *duration of the step* (*D*), which are represented graphically in Fig. 1. The *climatic staircase* begins at the year *S*, with several steps of constant duration *D*, to end at the year *E*. In the following, we will use equivalently “averaging period” or “duration of the step” for *D*. The constraint from the data set (http://data.giss.nasa.gov/gistemp/tabledata_v3/GLB.Ts+dSST.txt) is 1880 ≤ *S* ≤ 2013. We choose here to go the farthest possible within the time series, i.e. *E* = 2013. From the above-mentioned range of timescales usually involved in the averaging process, we choose 5 ≤ *D* ≤ 30. For both variables, we use discrete steps (1 yr), so that there are 134 discrete values for *S* and 26 values for *D*.

At first sight, one could think that the study zone is a rectangular zone *R* defined by 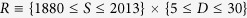 embracing the 134 × 26 = 3484 points. It is however not the case, as there exists an intrinsic constraint to the proposed hybrid method: at least two steps must exist in order to be able to perform the linear regression. For the consistency of the study, any incomplete step is indeed not considered. This defines an upper bound *S*_*max*_ for *S* (once *D* has been chosen). *S*_*max*_ is defined as the highest discrete value of *S* satisfying the inequality *S* + 2*D* ≤ 2013. Note that on the opposite side, the maximum number of steps is 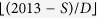, where the lower integer part of *n* is denoted by 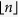.

**Figure 2:**
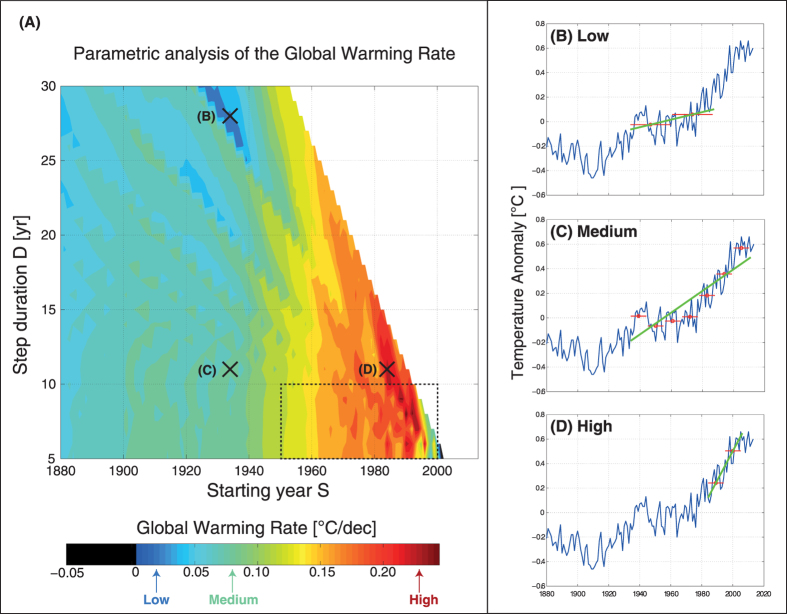
(**A**) Parametric analysis: a Global Warming Rate (GWR) value is computed by the hybrid method (Fig. 1.) for each (*S*, *D*) point of the grid (134 discrete values for *S* and 26 values for *D*), while *E* = 2013 is kept constant. The white triangular zone corresponds to a “forbidden zone”; there are 3029 values in the study zone. The dotted rectangle highlights the zone which is zoomed in on Fig. 3. Three reference samples are illustrated on the right: (**B**) Low GWR (+0.031 °C/dec), obtained for (*S*_*B*_, *D*_*B*_) = (1934, 27); (**C**) Medium GWR (+0.088 °C/dec), obtained for (*S*_*C*_, *D*_*C*_) = (1934, 11); (**D**) High GWR (+0.239 °C/dec), obtained for (*S*_*D*_, *D*_*D*_) = (1984, 11). There is an overall acceleration of the global warming *whatever the value of the averaging period D*. And 99.9% of the 3029 Earth’s climatic irregular staircases obtained by the hybrid method are rising.

That upper bound *S*_*max*_ creates a boundary which is clearly visible in Fig. 2: the “forbidden zone” *S* > *S*_*max*_ is the white triangular zone on the right, containing 455 discrete values. Hence the computation of the parametric analysis is finally made on 3484 − 455 = 3029 values of the (*S*, *D*) pair.

However the steps of such climatic staircases are rarely uniformly spaced, so that yet another question naturally arises: how to draw a single trend line accross such irregular staircases, in order to compute a warming rate? In the literature, linear trend lines are indeed usually applied on the time series *itself*, like it is done in [3, p. 5] in order to calculate a “warming of 0.85 [0.65 to 1.06] °C, over the period 1880 to 2012.” — but how to perform a linear regression on irregular staircases?

If we reduce each step to a point, by taking the midpoint of the step, then the problem is reduced to a classical linear regression problem between points, as shown in Fig. 1 — the way chosen to perform the linear regression is the least squares fitting.

This original method combining stairstep averages and linear regression by least squares fitting is called the *hybrid approach* and will be used herein. More precisely, the GWR obtained by the hybrid method is defined as the slope of the linear regression through the midpoints of the steps of constant duration *D*, starting from the year *S* and ending at the year *E*.

From the method itself, it is reasonable to think that some periodicity would appear in the results, due to the fact that shifting *S* by *D* would produce the same GWR. It is nevertheless not an exact periodicity, as that shift makes the staircase one step shorter on the left side. So, we will call it a *pseudo-periodicity* in the following.

We can make the same kind of reasoning for varying variables (*S*, *E*), while *D* is kept constant (we take *D* = 10 as a reference).

## Results and Discussion

In order to obtain the full picture, we perform the parametric analysis by allowing *S* and *D* to vary between their respective bounds, while *E* is fixed to 2013. The GWR is computed by means of the hybrid method (Fig. 1.) for each (*S*, *D*) point of the grid within the study zone (there are 3029 values). The results are represented in the contour plot (panel (A) of Fig. 2). For illustration purposes, we choose three reference samples shown in the three panels on the right of Fig. 2: (B) Low GWR (+0.031 °C/dec), obtained for (*S*_*B*_, *D*_*B*_) = (1934, 27); (C) Medium GWR (+0.088 °C/dec), obtained for (*S*_*C*_, *D*_*C*_) = (1934, 11); (D) High GWR (+0.239 °C/dec), obtained for (*S*_*D*_, *D*_*D*_) = (1984, 11).

First of all, one is struck by two features in the results, which are readily distinguishable on Fig. 2: vertical strips and diagonal structures. The diagonal structures are an artifact intrinsic to the method itself. Indeed, when looking attentively, one remarks that the spacing between the diagonal lines increases with *D*. More precisely, the distance between two successive minima or maxima for a line *D* = *cst* is exactly *D*. Hence it is clear that the diagonal structures are due to the above-mentioned *pseudo-periodicity*. This artifact is however not really embarrassing in order to interpret the results, as the other feature (vertical strips) is still clearly visible. The location of the extrema is also an information in itself.

The vertical strips (like the green, yellow, orange ones) visible on Fig. 2 have on the contrary an insightful meaning, and allow to draw sound conclusions. We observe first that the GWR has approximately the same value for any starting year *S*, *whatever the value of D*. In other words, the GWR is less dependent on *D* than on *S*. Secondly, the GWR is clearly an overall increasing function of the starting year *S*, except for the very last 15 years. It is clear from those vertical strips that there is an acceleration in global warming *whatever the value of D*. This conclusion generalizes the partial results often obtained for only a few periods of time in the literature (e.g. only five periods of time are considered in [3, Table 2.7, p. 193]).

This acceleration is especially marked after 1960, which is in concordance with the results mentioned in the last IPCC report AR5 [3, Table 2.7, p. 193]. In particular, Hansen^17^ gives a mean value of 0.065 °C/dec for the period 1880–2012, which is in accordance with the corresponding zone starting at *S* = 1880 in Fig. 2, yielding Medium GWR values. Hansen also gives the mean value 0.124 °C/dec for the period 1951–2012, and also 0.161 °C/dec for the period 1979–2012, which are again consistent with Medium-to-High values in Fig. 2. Moreover, the mean rate +0.17 °C/dec of the *regular* staircase (*S*_*R*_, *D*_*R*_) = (1970, 10) mentioned in the last IPCC report [3, Fig. SPM 1a), p. 6] for the three last decades, corresponds remarkably well with the zone starting at *S* = 1970 in Fig. 2 — the fact that the reference periods differ plays no role here.

From a meticulous inspection of panel (A) in Fig. 2, it can additionally be deduced that there is a relative slowdown in surface temperature rise, similarly to the literature^5^,^6^,^7^. More precisely, we can observe a green zone (+0.11 °C/dec) which begins at *S* = 1950 and a blue zone (+0.06 °C/dec) which begins at *S* = 1998. These zones are consistent with the following IPCC statement: “As one example, the rate of warming over the past 15 years (1998–2012; 0.05 [0.05 to 0.15] °C per decade), which begins with a strong El Niño, is smaller than the rate calculated since 1951 (1951–2012; 0.12 [0.08 to 0.14] °C per decade)” [3, SPM, p. 3]. In that reference, detailed values for trends for 15-year periods starting in 1995, 1996, and 1997 are also given (+0.13 °C/dec, +0.14 °C/dec and +0.07 °C/dec respectively).

But yet another conclusion can also be drawn from an even more meticulous inspection of panel (A) in Fig. 2. Only three GWR values are slightly negative (at the very end of the study zone, on the bottom right). A very powerful numeric lens is needed to access these. When sufficiently zoomed in, we find that the three corresponding pairs of parameters are (*S*_1_, *D*_1_) = (2001, 6), (*S*_2_, *D*_2_) = (2002, 5) and (*S*_3_, *D*_3_) = (2003, 5). One concludes that a slightly negative slope in global warming could only be achieved for the very last 10–15 years, and by considering only two-steps staircases of duration 5 or 6 yr. In other words, it is really cherrypicking: it can be found only at the 0.1% level (3/3029), which is clearly anecdotic — this research allows a very exact quantification of that level, on the basis of the full picture provided by the parametric analysis. Note that the conclusion holds when considering the other dataset HadCRUT4 (we find exactly the same three pairs of parameters yielding negative GWR).

In parallel, we note that the highest GWR values (GWR > 0.15 °C/dec) are easily achievable for most of the *D* range, except for *D* > 25, which leads us directly very far away in the past (at least 50 yr, since two steps are required for the hybrid method to work). We also note finally that the contour zones are more homogeneous towards high values of *D*, which seems logical. On the contrary, considering low values of *D* leads to heterogeneities. This is consistent with the statement “Due to natural variability, trends based on short records are very sensitive to the beginning and end dates and do not in general reflect long-term climate trends.” [3, SPM, p. 3].

**Figure 3:**
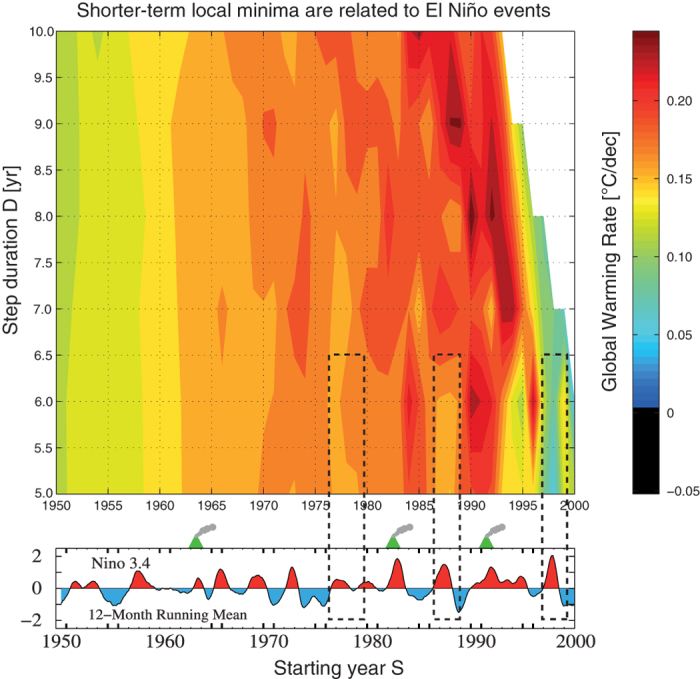
*(top)*: Zoom on the highlighted rectangle in Fig. 2. *(bottom)*: NINO34 index [17, Fig. 10] — used and modified with permission. The dashed rectangles highlight the correspondence between the shorter-term local minima of the parametric analysis and the warm phase of ENSO. This proves that choosing El Niño years as a starting year *S* gives lower global warming rates — except if there is a volcanic cooling in parallel.

Let us now consider what happens for lower values of *D* in the most recent years (Fig. 3). From the parametric analysis, we see that there is an alternation of GWR minima and maxima zones which is very clear for *D* = 5 and 6. Minima are located around *S* = 1977–78, 1986–87 and 1998–99. We immediately recognize these periods as they correspond to historical El Niños (http://www.elnino.noaa.gov/) — where 1997–1998 is a very strong El Niño (Super El Niño). The internal variability of climate has been analyzed in details by many authors [3, Box 2.5, Table 1, p. 233–235], who have proposed numerous indices like NIÑO3.4^19^ to describe it — since the seminal work of Trenberth about the definition of El Niño^19^.

So, we plotted in the bottom of Fig. 3 the NIÑO3.4 index — the panel has been modified from [17, Fig. 10]. The dashed rectangles highlight the correspondence between the shorter-term local minima of the parametric analysis and the warm phase of ENSO (water are warmer than usual in the Eastern Pacific). This Figure gives graphical evidence that choosing El Niño years as a starting year *S* yields lower global warming rates — except if there is a volcanic cooling in parallel (e.g. 1982–83 and 1991–92). The timescales of the two physical processes are indeed about the same order of magnitude (2–4 yr oscillations for ENSO and 5 yr global cooling for large volcanic eruptions such as El Chichón in 1982 or Pinatubo in 1991).

As mentioned in [Ref. 17], there exists a well-known strong correlation of global surface temperature with the Niño index^13^,^20^ (“The correlation is maximum (at 30%) with 12 month running mean global temperature lagging the Niño index by 4 months”). Hence, as the temperature record is higher for a value of *S* corresponding to an El Niño year, it is logical that the climatic staircase starting from there would then be less steep than staircases occurring on other times. On the opposite, La Niña years (like 1988–1989) correspond to GWR local maxima zones. In other words, El Niño clearly modulates the GWR obtained by the hybrid method. The statement for the last El Niños was already mentioned in [3, SPM, p. 3] for the least squares method, but we extend it to other periods in the general framework, and we also give a graphical representation.

Note that while it is clear that El Niño modulates the results by way of the variable *S* (which varies by 1 yr increments), the variable *D* also plays a role: the correspondence is indeed more pronounced over short-term horizons (*D* = 5 or 6 yr). The ENSO oscillating physical effect is indeed finally swiped out by the stairstep averaging process if we consider higher values of the averaging period *D* (*D* ≥ 7 yr).

**Figure 4:**
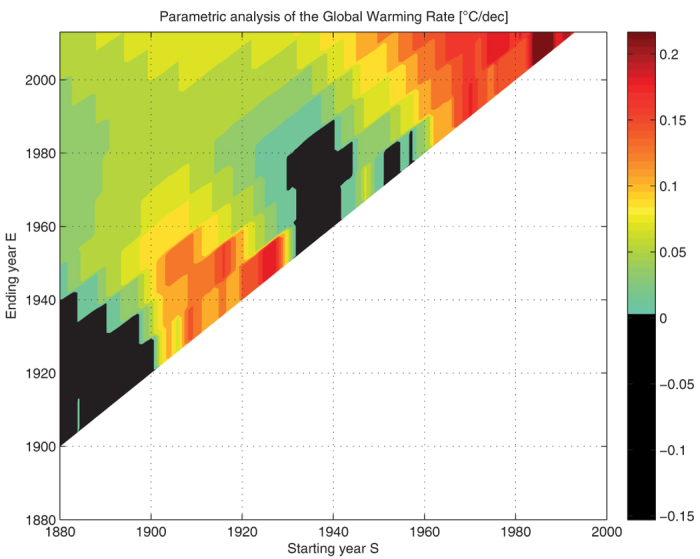
Parametric analysis: a GWR value is computed by the hybrid method (Fig. 1.) for each (*S*, *E*) point of the grid while *D* = 10 is kept constant. The white zone corresponds to a “forbidden zone” (the boundary is *E* = *S* + 20). Two black zones correspond to specific choices of (*S*, *E*) for which we have negative GWR: *S* taken on a relative maxima of the temperature curve, and considering a shorter horizon of time (i.e. near the diagonal).

In Fig. 4 we consider what happen now if we let both *S* and *E* vary (*D* is kept fixed at *D* = 10). First of all, there exists a boundary *E* = *S* + 20 which is intrisic to the method (there are minimum two steps). Second, there is some structure (vertical bands of length *D*), which is also due to the method, and which is logical, as only an integer number of steps has to be considered. Third, when the ending year *E* gets lower than 2013, two major zones with negative GWR can be accessed. The two black zones correspond logically with *S* around 1880 and 1940, which are relative maxima of the temperature curve. These zones are relatively large, as one can easily achieve negative slopes when considering shorter period of time (i.e. near the diagonal). This contrast with the case *E* = 2013 where only very few negative values (3) were observed.

One can now access the GWR for any period within the time frame of the data. For example, we can look at the period 1901–1950 (*S* = 1901, *E* = 1950). Accordance is found with the last IPCC Report AR5 [3, Table 2.7, p. 193] — our results agree with Hansen’s value 0.090 °C/dec.

## Conclusions and Perspectives

An original hybrid method for combining the stairstep averages with linear regression in order to derive a new type of climatic trend has been proposed. The so-obtained GWR is defined as the slope of the linear regression through the midpoints of steps of constant duration *D* (averaging period), starting from the year *S* and ending at year *E*. This methodology has been applied on the NASA GISS Land-Ocean Temperature Index (LOTI) time series and also on the HadCRUT4 data set, and the results are similar for both data sets. The effect of the two parameters (*S* and *D*) has been systematically studied in details, by allowing them to vary on a wide range (1880 ≤ *S* ≤ 2013 and 5 ≤ *D* ≤ 30). Hence the full picture could be accessed by this parametric analysis, uncovering a whole previously unexplored space. The study zone holds 3029 values of the Global Warming Rate (GWR), instead of only a couple of values for a reduced number of selected periods. The variations in the (*S*, *E*)-plane have also been studied.

It is the first time, to the best of our knowledge, that such a graphical view over such a wide parameter space has been given for the GWR, so that previous partial results are casted in a larger framework. In this paper, we found that there is an overall acceleration of the global warming *whatever the value of D*.

The GWR values found are in concordance with several results mentioned in the literature, but generalize them to a much wider space. In particular, accordance is found with last IPCC Report AR5 [3, Table 2.7, p. 193] — our results agree with Hansen’s values for the three periods 1880–2012, 1951–2012 and 1979–2012 (0.065 °C/dec, 0.124 °C/dec and 0.161 °C/dec respectively). The mean rate +0.17 °C/dec of the *regular* staircase (*S*_*R*_, *D*_*R*_) = (1970, 10) mentioned in the last IPCC report [3, Fig. SPM 1a), p. 6] for the three last decades, matches remarkably well the corresponding zone of our study.

A relative slowdown in surface temperature rise has been observed for the last 15 yr, similarly to the literature^5^,^6^,^7^ — but we have shown additionally that a slightly negative slope in global warming could only be achieved for the very last 10–15 years, and by considering only two-steps staircases of duration 5 or 6 yr. We also computed that it can be found only at the 0.1% level (3/3029), which is clearly anecdotic. Otherwise, 99.9% of the 3029 Earth’s climatic irregular staircases are rising.

We also give graphical evidence that choosing an El Niño year as a starting year *S* gives lower global warming rates — except if there is a volcanic cooling in parallel (e.g. 1982–83 and 1991–92). The statement for the last El Niños was already mentioned in [3, SPM, p. 3] for the least squares method, but we extend it to other periods in the general framework.

While we used mainly one single data set in this article for illustration purposes, this study has also been performed on another data set (HadCRUT4) used to estimate the GMST anomaly. No fundamental difference with the results presented herein has been noticed (the results are so similar that the graphs cannot be distinguished by eye), which was to be expected as both time series exhibit similar evolutions [3, Fig. 2.20, p. 193].

We emphasize the main conclusion of this study, i.e. that there is an overall *acceleration* of the global warming whatever the value of *D*. This conclusion is indeed of major importance regarding the increasing consequences that such a still increasing global warming rate will have. There is no doubt that there is already an impact on the whole planet. What remains still unknown is the exact extent of these increasing impacts on the living species — including the human beings —, on their health, and even on the sustainability of human society over the long term.

## Additional Information

**How to cite this article**: De Saedeleer, B. Climatic irregular staircases: generalized acceleration of global warming. *Sci. Rep.* **6**, 19881; doi: 10.1038/srep19881 (2016).

## References

[b1] Helm V., Humbert A. & Miller H. Elevation and elevation change of Greenland and Antarctica derived from CryoSat-2. The Cryosphere 8, 1539–1559 (2014).

[b2] Rignot E., Velicogna I., van den Broeke M. R., Monaghan A. & Lenaerts J. T. M. Acceleration of the contribution of the Greenland and Antarctic ice sheets to sea level rise. Geophys. Res. Lett. 38, L05503 (2011).

[b3] Stocker T. F. . *IPCC, 2013: Climate Change 2013: The Physical Science Basis. Contribution of Working Group I to the Fifth Assessment Report of the Intergovernmental Panel on Climate Change*. (Cambridge University Press, Cambridge, United Kingdom and New York, NY, USA, 1535 pp., 2013).

[b4] England M. H. . Recent intensification of wind-driven circulation in the Pacific and the ongoing warming hiatus. Nature Clim. Change 4, 222–227 (2014).

[b5] Huber M. & Knutti R. Natural variability, radiative forcing and climate response in the recent hiatus reconciled. Nature Geosci 7, 651–656 (2014).

[b6] Maher N., Gupta A. S. & England M. H. Drivers of decadal hiatus periods in the 20th and 21st centuries. Geophys. Res. Lett. 16, 5978–5986 (2014).

[b7] Guemas V., Doblas-Reyes F. J., Andreu-Burillo I. & Asif M. Retrospective prediction of the global warming slowdown in the past decade. Nature Clim. Change 3, 649–653 (2013).

[b8] Tollefson J. Climate change: The case of the missing heat. Nature 505, 276–278 (2014). 24429612 10.1038/505276a

[b9] Kintisch E. Is Atlantic holding Earth’s missing heat? Science 345, 860–861 (2014). 25146261 10.1126/science.345.6199.860

[b10] Balmaseda M. A., Trenberth K. E. & Källén E. Distinctive climate signals in reanalysis of global ocean heat content. Geophys. Res. Lett. 40, 1754–1759 (2013).

[b11] Nuccitelli D., Way R., Painting R., Church J. & Cook J. Comment on “Ocean heat content and Earth?s radiation imbalance. II. Relation to climate shifts”. Phys. Lett. A 376, 3466–3468 (2012).

[b12] Solomon S., Plattner G.-K., Knutti R. & Friedlingstein P. Irreversible climate change due to carbon dioxide emissions. Proc. Natl. Acad. Sci. USA 106, 1704–1709 (2009). 19179281 10.1073/pnas.0812721106 PMC2632717

[b13] Thompson D. W. J., Wallace J. M., Jones P. D. & Kennedy J. J. Identifying Signatures of Natural Climate Variability in Time Series of Global-Mean Surface Temperature: Methodology and Insights. J. Climate 22, 6120–6141 (2009).

[b14] Morice C. P., Kennedy J. J., Rayner N. A. & Jones P. D. Quantifying uncertainties in global and regional temperature change using an ensemble of observational estimates: The HadCRUT4 data set. J. Geophys. Res. 117, 22 (2012).

[b15] Vose R. S. . NOAA’s Merged Land–Ocean Surface Temperature Analysis. Bull. Amer. Meteor. Soc. 93, 1677–1685 (2012).

[b16] Chen X. & Tung K.-K. Varying planetary heat sink led to global-warming slowdown and acceleration. Science 345, 897–903 (2014). 25146282 10.1126/science.1254937

[b17] Hansen J., Ruedy R., Sato M. & Lo K. Global Surface Temperature Change. Rev. Geophys. 48, RG4004 (2010).

[b18] Hansen J. . Global temperature change. Proc. Natl. Acad. Sci. USA 103, 14288–14293 (2006). 17001018 10.1073/pnas.0606291103 PMC1576294

[b19] Trenberth K. E. The Definition of El Niño. Bull. Amer. Meteor. Soc. 78, 2771–2777 (1997).

[b20] Foster G. . Comment on “Influence of the Southern Oscillation on tropospheric temperature” by McLean J. D., de Freitas C. R. & Carter R. M. J. Geophys. Res. 115, D09110 (2010).

